# Intravascular Leiomyoma Considered Preoperatively as Uterine Sarcoma: A Rare Case

**DOI:** 10.1089/whr.2023.0091

**Published:** 2024-04-04

**Authors:** Yingyao Wang, Song Xu, Caibo Wang, Wenjuan Li, Jianhao Xu, Zhiwei Zhu, Qin Liu, Lixia Zhu

**Affiliations:** ^1^ Department of Gynecology, Kunshan Maternity and Children's Health Care Hospital, Suzhou, China.; ^2^ Department of Pathology, Affiliated Kunshan Hospital of Jiangsu University, Suzhou, China.; ^3^ Department of Gynaecology, Affiliated Kunshan Hospital of Jiangsu University, Suzhou, China.

**Keywords:** intravascular leiomyoma, uterine sarcoma, pathology

## Abstract

Intravascular leiomyoma (IVL) is usually defined as a histologically benign leiomyoma that originates in a uterine fibroid or the intrauterine vein wall and grows and expands intravenously. We report a case in which pelvic IVL was detected early and discuss the early diagnosis of and best treatment for this tumor.

## Introduction

The majority of the benign neoplasm occurring in the uterus are smooth muscle tumors and is the most common reason why women choose to undergo hysterectomy.^1,2^ Uterine fibroids are composed mainly of spindle-shaped smooth muscle cells and unequal amounts of fibrous connective tissue. The muscle cells were uniform in size, were arranged in a vortex or shed shape, and had rod-shaped nuclei. When uterine fibroids undergo degeneration, they lose their typical structure, often resulting in sarcomatous degeneration. Moreover, there are specific histological types of leiomyomas, such as epithelioid leiomyomas and intravenous and disseminated peritoneal leiomyomas.^3,4^ Due to their rarity, the nature and malignant potential of these specific leiomyomas have been poorly studied.

Among these, intravenous leiomyoma deserves attention. It is rarely discovered before surgery or when serious complications develop. Intravenous leiomyomas have benign histological features and rarely show mitotic figures but are biologically aggressive.^5–8^ It is characterized by the presence of vascular elongation and benign smooth muscle lesions invading the pelvic and systemic vascular systems in a worm-like manner.^9^ Most of these tumors are diagnosed by cardiologists when they extend into the inferior vena cava, right heart, or pulmonary artery.^10,11^ Nonspecific clinical features and variable imaging findings of intravenous leiomyoma, which makes preoperative diagnosis difficult.^12^ Therefore, it is rarely recognized and often misdiagnosed, causing patients to miss the opportunity for timely treatment. Here, we report the differential diagnosis and surgical methods used for rare intravenous leiomyomas. The patient has informed and consented that data about the case will be submitted for publication.

## Case Presentation

In April 2023, a 44-year-old woman experienced abdominal pain for 9 days and complained of worsening of her symptoms over 3 days. She had initially presented with similar abdominal pain a year prior; however, the patient was afraid to seek timely medical attention due to the COVID-19 pandemic. The patient had no relevant medical history except for one uneventful caesarean section delivery, and there was no family history of uterine fibroids. The gynecological examination showed that the patient had a 16-week- uterus, and fullness was felt in the bilateral adnexal region.

**FIG. 1.**
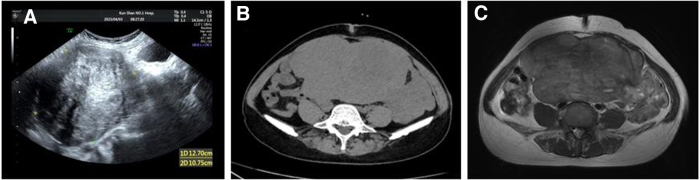
IVL radiographic image. **(A)** Transvaginal ultrasound was performed 3 days before surgery. **(B)** Axial CT scan of the abdomen showing a large pelvic mass. **(C)** MRI images showing a large mass of uterine origin that was initially considered a uterine sarcoma. CT, computed tomography; IVL, intravascular leiomyoma; MRI, magnetic resonance imaging.

Upon admission, transvaginal ultrasound revealed a large pelvic mass ∼15 × 12 × 10 cm in size, suggesting the presence of large uterine fibroids; moreover, there was no obvious uterine echo, the boundary was not clear, and the shape was regular. There were no significant changes in the bilateral ovaries (Fig. 1A). A computed tomography (CT) scan of the whole abdomen revealed a large soft tissue mass in the lower abdomen and pelvic cavity 100 × 170 mm in size with uneven density, strip calcification foci and flake low-density shadows that were connected to the uterus below (Fig. 1B). Pelvic magnetic resonance imaging (MRI) revealed a large pelvic mass with a size of 178 × 94 mm, which was considered to have originated from the uterus. There was a high possibility of myoma with malignant sarcomatous degeneration. A cystic lesion in the right adnexal area with a size of 22 × 18 mm was found (Fig. 1C). Tumor markers levels (AFP, CEA, CA199, CA125, CA153, and ferritin) were not elevated in the patient before surgery.

After counselling, the patient first underwent total abdominal hysterectomy and bilateral salpingectomy with preservation of both ovaries to prevent the effects of estrogen loss. During the operation, the uterus was as large as it was at 6 months of gestation and was covered with wadded cotton, additionally the surface was uneven, and the boundary was irregular. The bladder was dense and attached to the lower anterior wall of the uterus, the left tubal root was thickened and sausage-shaped, with a size of ∼15 × 6 cm; the right tubal root was also thickened, with a size of ∼3 × 4 cm. A mass resembling a cluster of grapes was found in both fallopian tubes. A purple–blue cystic mass with a diameter of ∼4 cm was found in the right ovary with a smooth surface. A cord-like mass was found around the left ovary extending into the suspended ligament of the left ovary, and the cord-like mass was palpable in the thickened suspended ligament of the ovary on both sides. The mass ran along the blood vessels, and the blood vessels were obviously dilated.

**FIG. 2.**
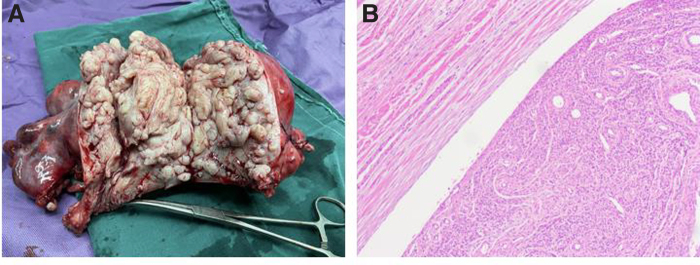
**(A)** Hysterectomy and double adnexectomy specimens. **(B)** The intravascular tumour almost entirely filled the lumens of the preexisting veins, as shown by H&E staining. H&E, haematoxylin and eosin.

Intraoperative frozen sections were reviewed, and a diagnosis of intravascular leiomyoma (IVL) was given. During macroscopic pathologic examination, the cut surface of the uterus showed ill-defined intramural nodules in the tumor (Fig. 2A). Bilateral oophorectomy and pelvic mass resection were performed intraoperatively after the patient's family was informed of the disease. No abnormalities were found on chest CT or echocardiography after the operation. The patient achieved a favor postsurgical recovery and was discharged after 7 days of hospitalization.

Under the microscope, the interwall tubercles of the uterus were observed to be composed of normal smooth muscle cells. These cells were benign, and showed no atypia or mitotic activity. The absence of morphologic features of malignancy was also noted. The tumor that was found in the irregular space of the uterine wall also invaded the lumen of the uterine vein. Foci of neoplastic leiomyomatous tissue sometimes entirely filled the lumens of the preexisting veins. Smooth muscle tissue could also be observed in the fallopian tubes and ovarian veins (Fig. 2B).

**FIG. 3.**
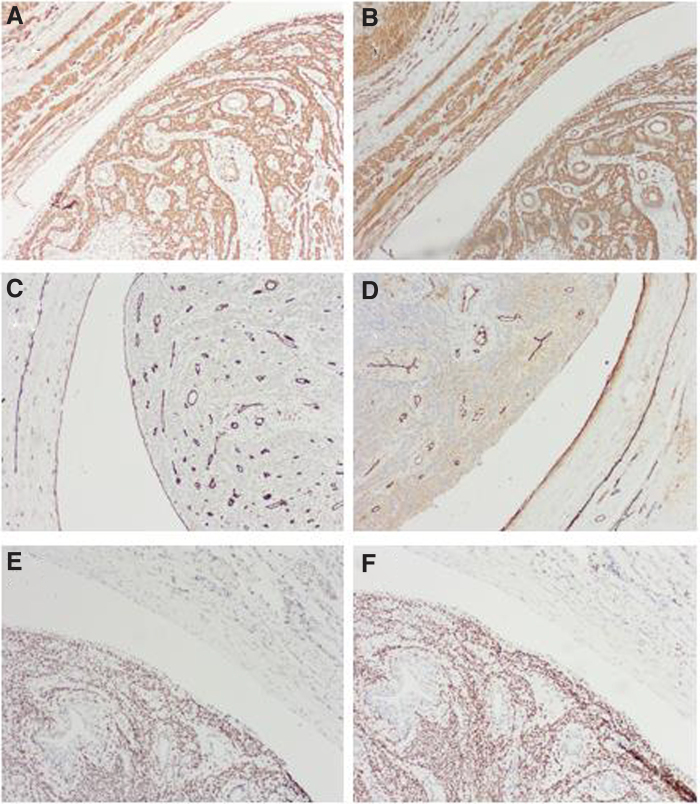
Histopathology of IVL. Immunohistochemical staining for calponin and smooth muscle actin demonstrated the myogenic structure of the lesion **(A, B)**. The endothelium of the blood vessels and the surface of the tumour are positive for CD31 and factor VIII-related antigen **(C, D)**. The IVL tumour was consistently positive for ER **(E)** and PR **(F)**. The original magnifications were all 100 × . ER, estrogen receptor; PR, progesterone receptor.

On immunohistochemistry, the tumor cells exhibited strong staining for calponin and smooth muscle actin (Figure 3A, B). CD31 and factor VIII-related antigen staining highlighted the endothelial cells of the blood vessels surrounding the foci of the leiomyoma that was intravascularly growing (Figure 3C, D). D2-40 and CD10 staining were negative. In other foci, the lesions were histologically identical to the intrauterine nodules. Immunohistochemical evaluation revealed that the area of the tumor that had developed intravascularly was positive for estrogen receptor and progesterone receptor (Figure 3E, F).

## Discussion

Uterine leiomyosarcoma is a malignant tumor that can metastasize to the blood, while venous leiomyomas have similarities.^13^ This case highlights the possibility of presenting similar to leiomyosarcoma, in which the tumor mass may extend significantly within the pelvic cavity but not progress elsewhere. It extends the current understanding but represents a diagnostic and therapeutic challenge. It was first described by Birsch-Hershfield in 1896.^4^ One study revealed that affected patients were predominantly women of reproductive age and often had no symptoms in the early stages.^14^

In 2016, Ma et al. clinically divided intravenous leiomyoma into four stages^15^: stage I: in which the tumor penetrates the uterine venous wall but is confined to the pelvic cavity; stage II: in which the tumor has expanded into the abdominal cavity but has not yet reached the renal veins; stage III: in which the tumor has reached the renal vein and inferior vena cava and extended further into the right atrium but has not reached the pulmonary artery; and stage IV: in which the tumor has reached the pulmonary artery, and/or lung metastasis is observed. Here, we report a patient with stage I disease in which early detection prevented further disease progression and decreased the risk. Prompt management is crucial for achieving good prognosis, as IVL can abruptly transition from asymptomatic to life-threatening, even sudden death.^16^

The optimal surgical method for and long-term prognosis of IVL are not fully understood.^8,12^ Yu et al. reported eight patients with intravenous leiomyomas at different clinical stages and collected surgical details and follow-up information.^17^ In agreement with the overwhelming majority of cases, complete surgical resection for any stages is beneficial if the patient is clinically able to undergo surgery.^18,19^

Due to the abundance of estrogen receptors in IVL cells, endogenous estrogen plays a role in relapse.^20,21^ Therefore, to reduce disease progression and the risk of future recurrence, it is not advisable to preserve the patient's ovaries in some cases where complete removal is not possible. The surgical treatments used for IVL of the female reproductive system include total hysterectomy, double adjunctive resection, and extrauterine tumor resection.^7,22^ The recurrence rate of patients whose leiomyoma lesions in the external uterine vein cannot be completely removed decreases after the removal of the uterus and ovaries.^5^ Because of the absence of special imaging findings, preoperative diagnosis is often challenging, such as uterine sarcoma and tumors are often considered as malignant tumors, such as uterine sarcoma.

In this case, preoperative imaging examination revealed the possibility of sarcomatous degeneration, and based on this, a diagnosis and treatment plan were created. After considering IVL of the uterus during the operation, we adopted the same treatment method used in most of the articles. Years of follow-up are needed after surgery, including regular assessment of the pelvic cavity and heart, major vessel color ultrasound, MRI, venography, and timely detection and treatment of recurrent residual lesions. For patients with postoperative recurrence, active surgery should be performed again.

At present, there are two main ideas about the origin of IVL. The first view is that IVL originate from venous vascular smooth muscle cells, while the other view is that IVL originate from uterine leiomyomas, which grow and form after invading the uterine wall veins.^5,23^ Our cases add more evidence to support the second one, due to the different immunostaining pattern between IVL and vascular smooth muscle cells. In recent years, there have been many studies on the pathogenic genes involved in this disease, and a variety of genes related to disease on set and relapse after treatment have been found.^24^ Such genetic research is expected to lead to the establishment of effective targeted therapies for this disease, control of the main lesion in the early stage of disease diagnosis, reduce the recurrence rate after surgery, and prolong the survival of patients.

## Abbreviations Used

CT: computed tomography
ER: estrogen receptor
H&E: hematoxylin and eosin
IVL: intravascular leiomyoma
MRI: magnetic resonance imaging
PR: progesterone receptor
